# CaMKIIβ Signaling drives expression of metabolic and stress response genes in skeletal muscle, and its loss contributes to the LGMDR1 phenotype

**DOI:** 10.1093/hmg/ddag090

**Published:** 2026-09-11

**Authors:** Irina Kramerova, Frieda Anastopulos, Diana Becerra, Ruby Goldstein de Salazar, Alexander R Keeble, Jeffrey S Chamberlain, Steven A Moore, Elizabeth M McNally, Melissa J Spencer

**Affiliations:** Department of Neurology, David Geffen School of Medicine at UCLA, University of California, 635 Charles E. Young Drive, Los Angeles, CA 90095, United States; Department of Neurology, David Geffen School of Medicine at UCLA, University of California, 635 Charles E. Young Drive, Los Angeles, CA 90095, United States; Department of Neurology, David Geffen School of Medicine at UCLA, University of California, 635 Charles E. Young Drive, Los Angeles, CA 90095, United States; Department of Neurology, David Geffen School of Medicine at UCLA, University of California, 635 Charles E. Young Drive, Los Angeles, CA 90095, United States; Center for Genetic Medicine, Northwestern University Feinberg School of Medicine, 303 E. Superior St, SQ 5-516 Chicago IL 60611, United States; University of Washington, 850 Republican Street, Seattle WA 98109, United States; Department of Pathology, University of Iowa, 200 Hawkins Drive, Iowa City, IA 52242, United States; Center for Genetic Medicine, Northwestern University Feinberg School of Medicine, 303 E. Superior St, SQ 5-516 Chicago IL 60611, United States; Department of Neurology, David Geffen School of Medicine at UCLA, University of California, 635 Charles E. Young Drive, Los Angeles, CA 90095, United States; Molecular Biology Institute, University of California, 611 Charles E. Young Dr., Los Angeles, CA 90095, United States; Jonsson Comprehensive Cancer Center, University of California, 10833 Le Conte Ave, Los Angeles, CA 90024, United States; Eli and Edyth Broad Stem Cell Center, University of California, 610 Charles E. Young Dr., Los Angeles, CA 90095, United States

**Keywords:** mouse model, muscular dystrophy, gene expression, disease pathways

## Abstract

Calcium calmodulin kinase II (CaMKII) signaling is activated by muscle use and drives gene expression that promotes slow oxidative muscle phenotype. Previously, we showed that the calpain 3 knockout (C3KO) mouse model of limb girdle muscular dystrophy R1 (LGMDR1) exhibits impaired slow-oxidative gene expression and these deficits were associated with reduced levels of the CaMKIIβ isoform and attenuated CaMKII signaling in C3KO muscles. To investigate the contribution of CaMKIIβ signaling to the calpain 3-deficient phenotype, we generated both loss-of-function (muscle-specific conditional knockout, *Camk2b* cKO) and gain-of-function (muscle-specific overexpression of a constitutively active CaMK2b) models. *Camk2b* cKO muscles exhibited myopathic features and weakness and their muscles failed to upregulate genes that promote oxidative metabolism and stress-responses following endurance exercise, similarly to C3KO muscles. Mitochondrial respiration showed reduced activity of complex I, complex II and fatty acid oxidation. RNA sequencing of LGMDR1 patient biopsies revealed a similar reduction in genes involved in oxidative metabolism, aligning human and mouse findings. Overexpression of a constitutively active *Camk2b* in C3KO muscles enhanced oxidative metabolism and improved functional performance. Our results support the hypothesis that blunted CaMKIIβ signaling contributes to the failed upregulation of genes involved in oxidative metabolism and stress-responses in C3KO muscles. These studies highlight metabolic insufficiency as a central contributor to LGMDR1 pathogenesis and support the therapeutic potential of targeting CaMKII signaling to ameliorate disease features in LGMDR1.

## Introduction

Skeletal muscles can sense functional demands and adapt to them by altering gene expression patterns that facilitate muscle remodeling. Depending on the type and duration of muscle activity, various signaling pathways are activated that result in adaptive transcriptional responses and lead to changes in muscle fiber phenotype [1–3]. Skeletal muscle myofibers are classified based on their contractile and metabolic properties into three main types: slow-oxidative (SO), fast-glycolytic (FG), and fast-oxidative glycolytic (FOG) [4]. Slow-oxidative fibers (Type I) rely primarily on oxidative metabolism and are slow to fatigue. Fast-glycolytic fibers (Type IIB) depend mainly on glycolytic metabolism, and fatigue quickly. Fast-oxidative glycolytic fibers (Type IIA and IIX) have intermediate properties, combining aspects of both oxidative and glycolytic metabolism. The type of myosin heavy chain expressed in a fiber (fast or slow) determines whether a fiber is Type I or Type II and dictates its twitch speed (slow or fast) and force generating capacity. In healthy muscle, the metabolic profile generally correlates with its myosin type, though FOG fibers show intermediate characteristics [4]. It is well established that different types of exercise training can change the fiber type and metabolic composition of a muscle. For example, endurance training results in a higher abundance of slow-twitch oxidative type fibers while resistance power exercise increases the content of fast-twitch glycolytic type IIA and IIX fibers [5]. These phenotypic changes affect the muscle’s myofibrillar protein composition, mitochondrial content and metabolic profile.

Ca^2+^ plays a vital role in muscle contraction and in activation of signaling pathways that regulate muscle adaptation [1]. Three major Ca^2+^-dependent signaling pathways are present in skeletal muscle that operate through either protein kinase C, calcineurin-NFAT and Ca^2+^-calmodulin dependent kinases II and IV (CaMKII and CaMKIV). All three pathways ultimately control the activity of transcription factors that promote transcription of genes of the slow oxidative phenotype. Such transcription factors include MEF2 and the transcriptional co-activator PGC1α [2].

In our previous studies we demonstrated that muscles from a mouse model of limb girdle muscular dystrophy R1 (LGMDR1, formerly known as LGMD2A) exhibit blunted Ca^2+^ release and reduced CaMKIIβ signaling [6–9]. LGMDR1 is one of the most common subtypes of LGMD and it is caused by mutations in the *CAPN3* gene encoding calpain 3 proteinase [10]. Clinically, symptoms typically begin in the second decade of life, and most patients lose ambulation within a decade of diagnosis [11]. Muscle biopsies from LGMDR1 patients show a mild dystrophic picture and mitochondrial abnormalities [12–14].

CAPN3 is a member of a large family of Ca^2+^-activated proteinases (calpains) that include both ubiquitously expressed and tissue-specific members. Pathogenic *CAPN3* mutations can impact both proteolytic and non-proteolytic functions of CAPN3 [15, 16]. CAPN3 is primarily expressed in skeletal muscle with low expression in the brain and no expression in the heart [17]. We previously demonstrated that *Capn3* knockout mice (C3KO) show a very mild dystrophic phenotype and that they replicate many features of LGMDR1 such as decreased myofiber size, abnormal cytoarchitecture of myofibers, and mitochondrial dysfunction [7, 18, 19].

Cell fractionation studies have revealed that CAPN3 is present in multiple subcellular compartments, suggesting that it plays different roles depending on its subcellular location [7, 20]. The majority of CAPN3 is found in the myofibrillar fraction, where it is anchored on titin at the N2 line [21]. Mutations disrupting this interaction are pathogenic, suggesting its vital importance for regulating calpain 3 activity and stability [18, 22, 23]. Within the sarcomere CAPN3 is believed to facilitate sarcomeric protein turnover during muscle remodeling [20, 24]. CAPN3 is also present in the membrane fraction where it is enriched at skeletal muscle triads, which are specialized structures where excitation-dependent Ca^2+^ release occurs [7, 25]. The triad invaginates at the sarcomere N2 line, which is also the site of CAPN3 binding on titin. Loss of CAPN3 in mouse muscle leads to decreased Ca^2+^ release, reduced abundance of triad-associated proteins, including Ryanodine Receptor I, and reductions in a specific isoform of CaMKIIβ that localizes to the triad complex [7, 26]. In subsequent studies we observed markedly reduced levels and activation of CaMKIIβ in C3KO muscles. By contrast, other signaling pathways, including AKT and AMPK, remain unaffected [8, 27]. The reduction in CaMKIIβ signaling in response to mechanical loading is associated with failure to upregulate slow oxidative and stress-response genes, impaired lipid metabolism, and a diminished capacity for endurance exercise [6, 8, 28].

While the reduction in CaMKII signaling aligns with abnormal changes in muscle remodeling and gene expression, the precise contribution of deficient CaMKII signaling, specifically mediated by the CaMKIIβ isoform, remains unclear. To investigate how loss of CaMKIIβ in skeletal muscle contributes to the LGMDR1 phenotype, we generated a loss-of-function model of *Camk2b* in skeletal muscle (*Camk2b* cKO). We examined how CaMKIIβ deficiency affects muscle morphology, gene expression profiles, and mitochondrial respiration to understand the contribution of CaMKIIβ signaling to the LGMDR1 phenotype. To determine the therapeutic potential of targeting this pathway, we overexpressed constitutively active CaMKIIβ in skeletal muscle and demonstrated that key features of the LGMDR1 phenotype were ameliorated. Together, these findings indicate that metabolic dysfunction is an under-recognized contributor to LGMDR1 pathogenesis, and that enhancing CaMKIIβ signaling is a potential therapeutic strategy for this disease.

## Results

### Muscle specific *Camk2b* cKO mice show a mild myopathy and muscle weakness

To generate *Camk2b* cKO mice, *Camk2b*^fl/fl^ mice [29] were crossed to mice carrying Cre recombinase under control of the human skeletal actin promoter (HSA). As shown in Fig. 1A, Cre expression led to specific and efficient knockout of the *Camk2b* gene in skeletal muscles. Levels of CaMKIIβ proteins were undetectable by western blot analysis of *Camk2b* cKO skeletal muscles (Fig. 1B, Supplemental Fig. 1). Of note, western blot analysis was performed for all mice used in experiments to ensure efficient knockout of CaMK2b (Supplemental Fig. 1 shows an example of such analysis in mice used for gene expression studies).

**Figure 1:**
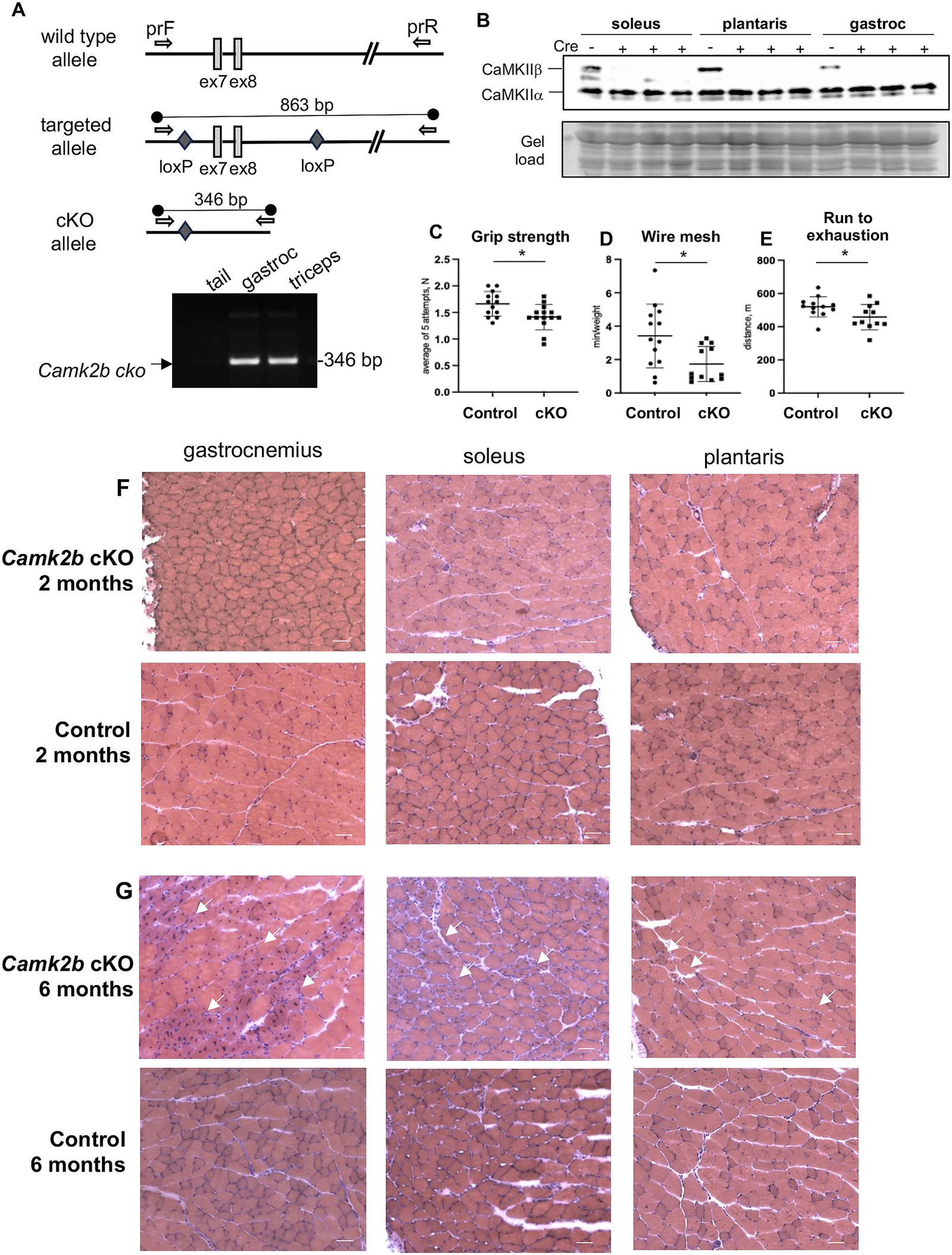
Functional assessments and histological evaluation of *Camk2b* cKO mice. (A) Schematic showing position of primers prF and prR used for genotyping of the *Camk2b* gene (upper panel). Genotyping PCR showing the cKO-specific band (346 bp) present in skeletal muscle DNA but not in tail DNA from Cre + mice (lower panel) (adapted from reference [29]). (B) Western blot analysis of whole muscle extracts of soleus, plantaris and gastrocnemius muscles using a CaMKII antibody that recognizes CaMKIIα and CaMKIIβ in skeletal muscles. A Cre-negative littermate was used as a control for each muscle type. Lanes marked with plus signs show Cre-recombinase positive mice. (C) Graphs of grip strength testing data from 6–7 month old male mice. The data show the average grip strength from 5 attempts in newtons (N). (D) Graph of wire mesh four limb hang test of 6–7 month old male mice showing the average hang time normalized by body weight. (E) Graph of run-to-exhaustion data from 6–7 month-old male mice showing the maximum time on the treadmill. Student’s *t-*test was used for statistical analysis; asterisks indicate statistical significance, *P* < 0.05; error bars represent standard deviation. (F and G) Representative hematoxilin and eosin staining of *Camk2b* cKO and control gastrocnemius, soleus and plantaris muscles from 2-month old (F) and 6-month old (G) mice. White arrows point to fibers with internalized nuclei. White bar, 50 mμ.

We next examined the effect of *Camk2b* ablation on muscle functional performance. The performance of *Camk2b* cKO mice was compared with their Cre-negative littermates in three functional tests, forelimb grip strength, wire mesh hang test and run to exhaustion. These tests evaluate the strength and fatigability of different muscle groups. As shown in Fig. 1C–E, *Camk2b* cKO mice showed significantly impaired performance in all three tests. These data suggest that loss of CaMKIIβ in skeletal muscle leads to muscle weakness and that the beta isoform has unique and essential functions in skeletal muscle that cannot be fully compensated for by other CaMKII isoforms.

Histological analysis of *Camk2b* cKO and control muscles was performed at 4 weeks, 2 months, 4 months and 6 months of age (Fig. 1F and G and Supplemental Fig. 2) using hematoxylin and eosin staining. A mild dystrophic phenotype was evident, characterized by a low frequency of fibers with internalized nuclei. On average, the percent of mislocalized nuclei did not exceed 1%; however, on rare occasions we observed clusters of fibers with mislocalized nuclei (an example is shown in Fig. 1G left panel). We immunostained muscles at 3 different ages (4 weeks, 2 months, and 6 months) for the macrophage marker CD68 and for developmental MyHC as a marker of regeneration (an example of such staining is shown of Supplemental Fig. 2B). We did not detect significant infiltration of the CD68-positive immune cells nor the presence of developmental MyHC-positive regenerating fibers. Dystrophin-deficient *mdx* muscles served as a positive control for staining (Supplemental Fig. 2C). These observations reflects the mild pathology observed in *Camk2b* cKO muscles, which is similar to C3KO muscles [18] and pre-sympotmatic LGMDR1 patients [30].

Since *CaMK2b* cKO mice showed significantly reduced performance (Fig. 1C–E) without any severe histological abnormalities, we hypothesized that reduced muscle performance could be due to metabolic deficiencies. Mitochondrial respiration was evaluated by Seahorse, and it was determined that the maximal oxidative rate was significantly reduced in *CaMK2b* cKO plantaris muscles compared to controls (Fig. 2A). Fiber type analysis based on the expression of different myosin heavy chain (MyHC) isoforms in plantaris of the same animals, did not reveal significant differences; however, a trend toward an increased percent of fast glycolytic type IIb fibers was observed in mice lacking CaMKIIβ (Fig. 2B). These results support a role for CaMKIIβ in promotion of oxidative metabolism in skeletal muscle and suggest that reduced functional performance of *CaMK2b* cKO mice could be due to energy deficits.

**Figure 2:**
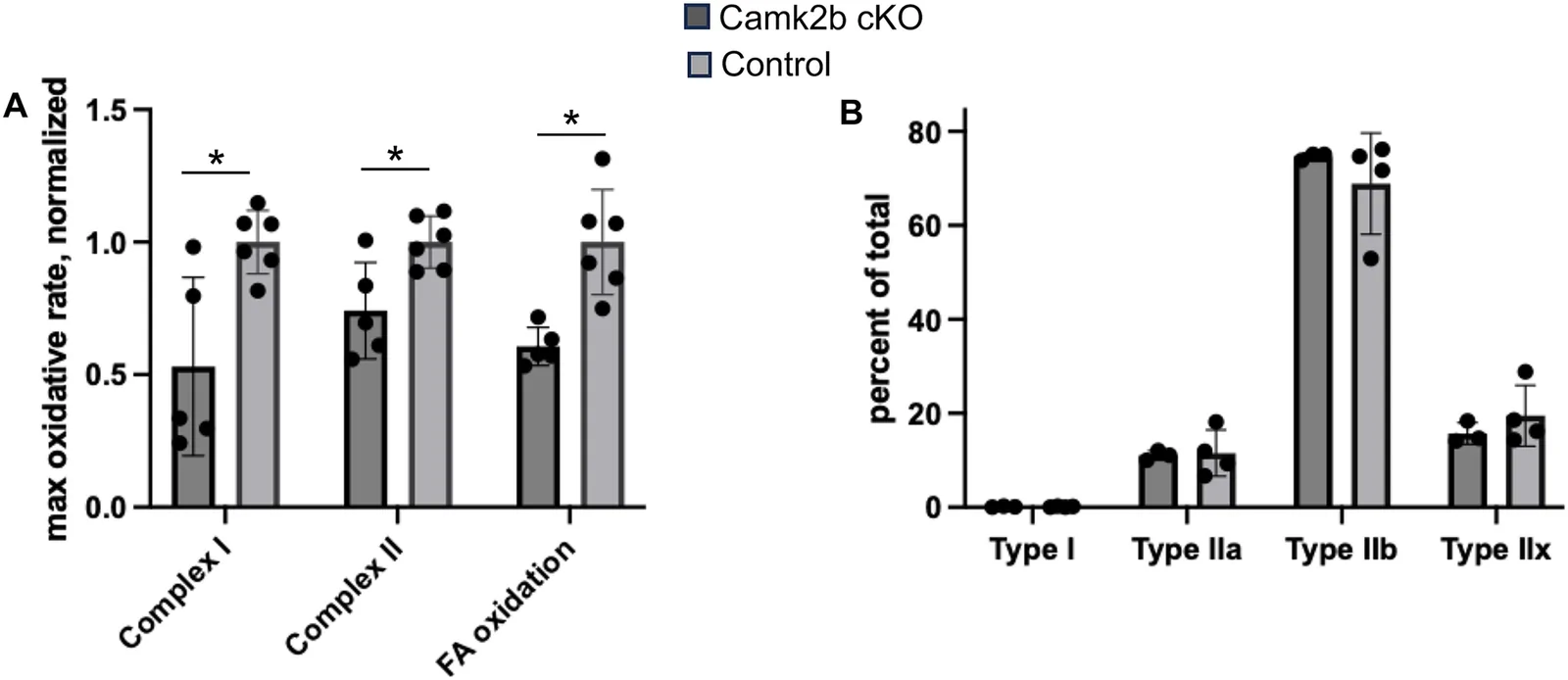
*Camk2b* cKO muscles show decreased oxidative capacity. (A) Mitochondrial respiration was assayed by Seahorse in mitochondrial fractions from freshly dissected plantaris muscles from 4-month-old *Camk2b* cKO and control mice. Maximal oxidative rate was determined using three different substrates to assess complex I, complex II, and fatty acid oxidation. The average of control muscles was set as one. (B) Fiber type analysis of the second plantaris muscles from the same mice. Asterisks indicate significant difference (*P* < 0.05) by two tailed *t-*test.

### Muscles of *Camk2b* cKO mice show impaired transcription of genes involved in oxidative metabolism

Prior studies in *Capn3* knockout mice (C3KO) demonstrated impaired muscle remodeling in C3KO after treadmill exercise or hindlimb unloading and reloading [8, 28]. In both models these changes coincided with blunted CaMKIIβ signaling and failure to activate a normal transcriptional response. Three categories of genes were not properly induced in C3KO mice after treadmill exercise: 1) genes encoding slow isoforms of myofibrillar proteins, 2) genes involved in mitochondrial and lipid metabolism and 3) stress response genes [8, 28].

In the current experiments, we used the identical 5 day-running protocol [8] to determine which impaired transcriptional programs could be specifically attributed to aberrant CaMKIIβ signaling. Male *Camk2b* cKO mice and their control littermates (n = 6 per group) were subjected to 1 h a day of treadmill running for 5 days. This protocol involved running at a moderate speed and was well tolerated by all mice, ensuring consistent exercise exposure across all animals. On the final day of running, gene expression was evaluated by bulk RNA-seq in plantaris muscles of 4 different groups of mice (*Camk2b* cKO sedentary, *Camk2b* cKO exercised, Cre^−^ control littermates sedentary and Cre^−^ control littermates exercised). Plantaris muscle was used to enable comparison with our prior studies. Histological assessments of plantaris muscles did not reveal significant morphological changes that would suggest that muscle damage occurred in response to running exercise (Supplemental Fig. 3).

To identify changes in gene expression in response to exercise, we compared transcriptomes of exercised vs sedentary muscles for each genotype. These comparisons revealed a similar pattern of failed upregulation of genes involved in oxidative metabolism in *Camk2b* cKO and C3KO mice (RNA-seq data for C3KO mice were previously reported in [8]). As shown in Fig. 3A, four of the ten top exercise-induced canonical pathways in control mice were associated with oxidative metabolism, including PPAR signaling, Apelin signaling, adipogenesis and protein kinase A signaling. In exercised *Camk2b* cKO mice, three of these four pathways failed to be upregulated, and although adipogenesis appeared increased, the magnitude of induction was substantially lower than in WT mice. A heatmap (Fig. 3B) further illustrates this pattern: many of the affected genes encode proteins central to lipid metabolism [31] such as lipoprotein lipase, (*Lpl*), a key regulator of free fatty acid release, proteins associated with lipid droplets (perilipins *Plin3* and *Plin4*), and enzymes involved in intracellular lipid metabolism such as diacylglycerol acyltransferase, *Dgat2*. Figure 3C provides an example of this blunted transcriptional response, highlighting reduced induction of select lipid metabolism genes in the cKO muscles compared to controls. Table 1 lists metabolic genes that are similarly dysregulated in both *Camk2b* cKO and C3KO muscle following exercise. Together, these data show that impaired induction of oxidative-metabolism pathways previously observed in C3KO mice can be at least partially attributed to blunted CaMKIIβ signaling.

**Figure 3:**
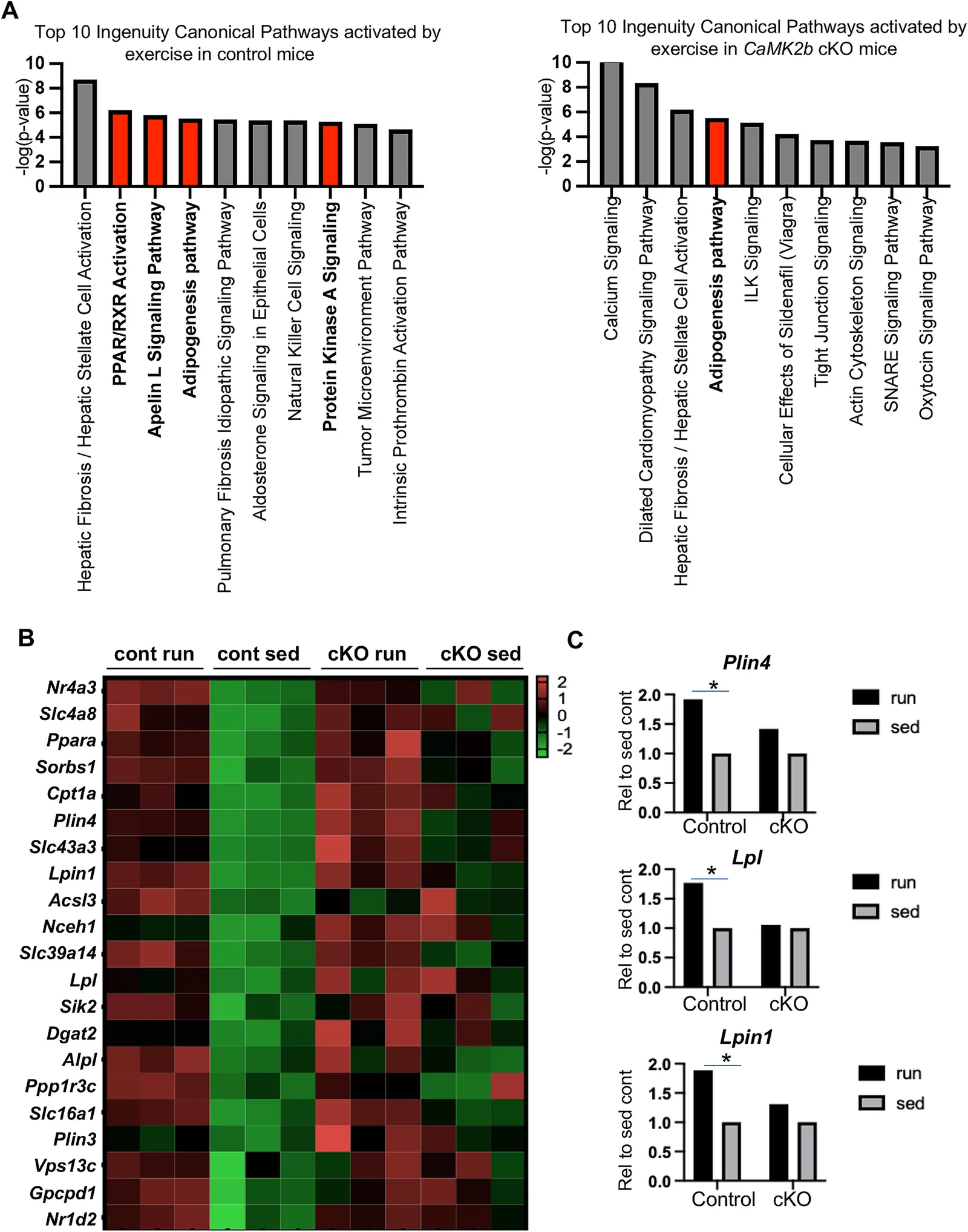
Reduced activation of metabolic genes in *Camk2b* cKO mice following five days of endurance (running) exercise. (A) Comparison of the top ten canonical pathways induced by running in control muscles (left panel) vs *Camk2b* cKO muscles (B) heat map of RNA seq data of plantaris from Cre-negative sedentary mice (cont sed), *Camk2b* cKO sedentary mice (cKO sed), Cre-negative mice after 5 days of running (cont run), and *Camk2b* cKO mice after 5 days of running (cKO run). All genes in the heat map were significantly up-regulated (*P* < 0.05) by exercise in control Cre-negative mice. All mice were males, 6–7-month-old. Color scale on the right shows z score. (C) Examples of the relative upregulation of the individual genes after running exercise. Level of expression in the sedentary control muscle for each genotype was set as 1. These genes were significantly different (*P* < 0.05) between control sed and control run muscles as indicated by the asterisk.

**Table 1:** Both CaMK2b cKO and calpain 3 knock out (C3KO) muscles show a similar inability to induce genes of lipid metabolism in response to exercise.

	C57/BL6	C3KO	Ratio of C57/C3KO	Cre− control	Cre + CaMK2b cKO	Ratio Cre−/Cre+
Ppara	1.67	0.98	**1.70**	2.44	1.55	**1.57**
Sorbs1	1.56	1.25	**1.25**	2.14	1.5	**1.43**
Plin4	1.47	0.92	**1.60**	1.92	1.42	**1.35**
Lpl	1.60	0/87	**1.84**	1.77	1.05	**169**
Sik2	1.29	085	**1.51**	1.73	1.18	**1.47**
Dgat2	2.27	1.04	**2.18**	1.72	1.40	**1.23**
Cd36	1.48	0.92	**1.61**	1.44	0.96	**1.50**
Per2	1.87	1.13	**1.65**	6.53	2.69	**2.43**
Hceh1	1.70	1.02	**1.67**	1.84	1.16	**1.59**
Bdh1	2.68	1.02	**2.63**	3.36	2.29	**1.47**

The table shows the ratio of gene expression in plantaris from C3KO (reported previously by Kramerova *et al*. 2016) [8] and Camk2b cKO mice as compared to their age matched control mice (C57/BL6 for C3KO and Cre-negative littermate controls for Camk2b cKO). The ratio represents the fold change in gene expression for C57 BL6 WT mice versus C3KO or for Cre-negative littermates versus Camk2b cKO mice (in bold).

In healthy muscles, endurance exercise up-regulates genes that encode slow muscle-specific isoforms of sarcomeric proteins. In our previous studies in C3KO mice, we showed that several sarcomeric genes were not properly up-regulated with exercise [8]. In contrast, these changes were not observed in exercised *Camk2b* cKO muscles. For example, in C3KO plantaris muscles, expression of *Myl2, Ckmt2* and *Myom3* were not induced by exercise [8] while the same genes were induced in exercised muscles from *Camk2b* cKO mice (Supplemental Fig. 4). These data suggest that the observed failure to upregulate slow sarcomeric genes in C3KO cannot be attributed to blunted CaMKIIβ signaling.

Increased muscle loading places mechanical stresses on the muscle and an adequate systemic response is required to prevent muscle damage and orchestrate regeneration. One of the important stress-induced molecules that can trigger a systemic response is a secreted form of the heat shock protein HSP70 [32, 33]. In our previous studies we found that secreted HSP70 (sHSP70), encoded by *Hspa1a* and *Hspa1b* genes, was highly up-regulated in WT mice in two different muscle adaptation protocols (running exercise and muscle reloading after a period of disuse). At the same time, no such up-regulation occurred in C3KO mice [28]. We also provided evidence that CaMKII signaling controls expression of sHSP70 in response to oxidative stress in C2C12 muscle cells [28].

*Camk2b* cKO muscles failed to upregulate both *Hspa1a* and *Hspa1b* following exercise. *Hspa1a* and *Hspa1b* transcript levels were 2.4 and 5.7 times higher in exercised control muscles compared to exercised *Camk2b* cKO muscles. It is noteworthy that several other stress response genes also failed to induce in *Camk2b* cKO mice compared to control littermates in response to exercise (Fig. 4A). These observations were confirmed by western blot analysis of sHSP70 and HSP90 in plantaris muscles from sedentary controls and exercised mice (Fig. 4 B-D). These data strongly suggest that CaMKIIβ-mediated signaling is necessary for induction of stress response genes in skeletal muscles and this may be one way in which mechanical loading is translated to biochemical signals.

**Figure 4:**
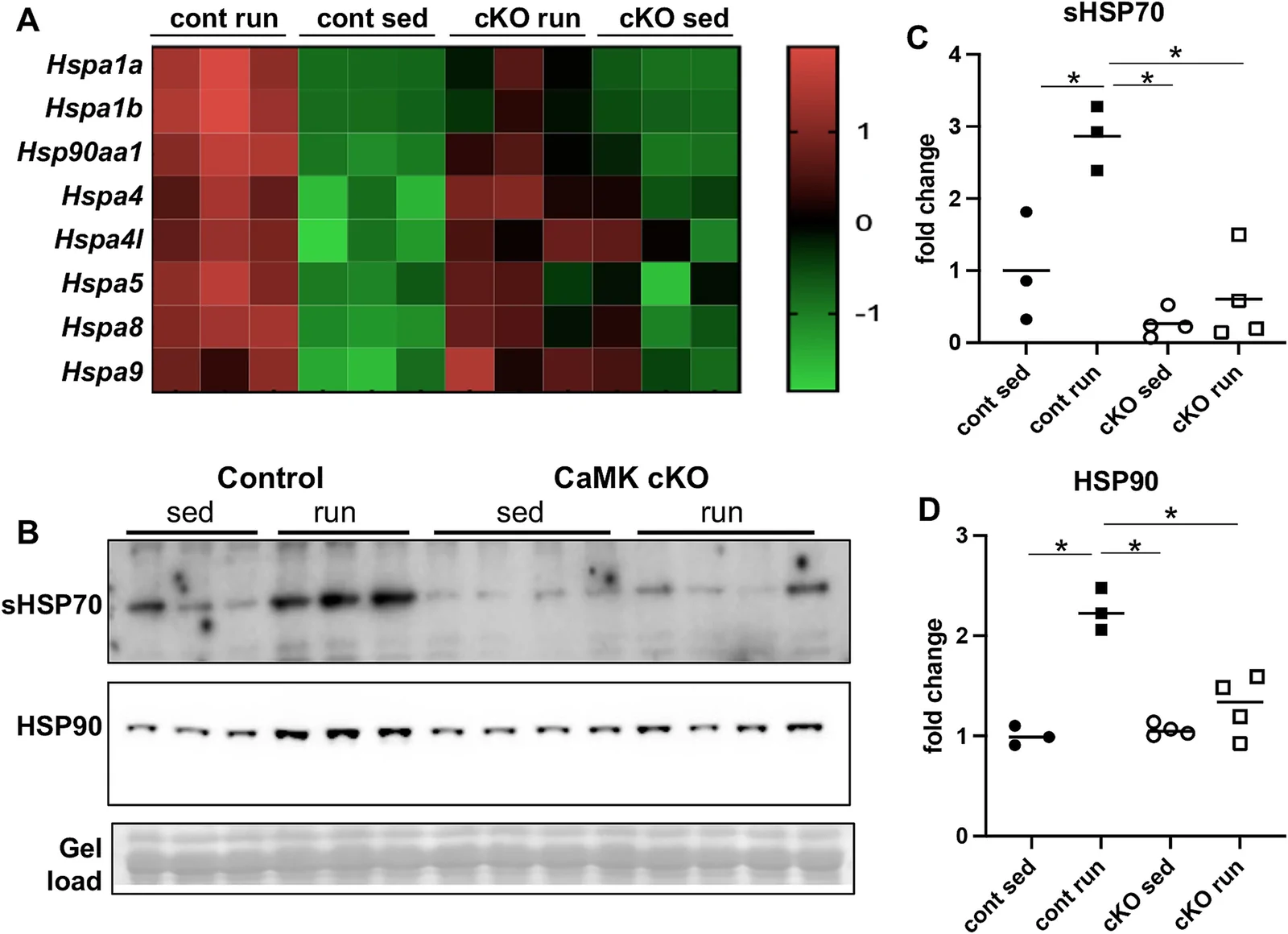
*Camk2b* cKO mice fail to induce heat shock protein gene expression after exercise. (A) Heat map of bulk RNAseq data showing expression of *HSP* genes in plantaris muscles from control vs *Camk2b* cKO mice. The genes shown were significantly up-regulated (*P* < 0.05) with exercise in control Cre-negative mice but not increased in *Camk2b* cKO mice. Color scale on the right shows z scores. (B) Western blot analysis of whole plantaris muscle extracts probed with anti-HSP70 (inducible) or HSP90 antibodies; (C and D) Quantitative analysis of the western blots shown in (B); error bars represent standard deviation; asterisks indicate significant difference (*P* < 0.05) by one-way ANOVA.

### Downregulation of genes involved in oxidative metabolism in LGMDR1 biopsies

To determine whether human LGMDR1 muscle biopsies exhibit gene expression changes similar to those observed in our mouse model, we performed bulk RNA sequencing on quadriceps biopsies from young LGMDR1 patients (17–22 years old) and control subjects (17–34 years old). This age range was selected to minimize confounding effects of advanced disease progression. RNA-seq analysis showed no major differences between LGMDR1 and healthy subjects in the expression of myofibrillar genes. In contrast, transcripts involved in oxidative metabolism and cellular stress responses were downregulated in LGMDR1 biopsies compared to healthy subjects (Tables 2 and 3 and Fig. 5). Metascape analysis further highlighted significant enrichment of pathways governing oxidative and lipid metabolism, all of which were reduced in LGMDR1 muscles. Notably, expression of *PPARGC1A* and *PPARGC1B,* encoding the metabolic transcriptional co-activators PGC1α and β, respectively, was diminished in patient samples. PGC1α is a principal regulator of mitochondrial biogenesis that promotes remodeling of muscle fibers towards an oxidative phenotype [2]. Consistent with our findings in the C3KO mouse model, several lipid-handling genes (*PLIN2, PLIN5, FABP3*) were less abundant in LGMDR1 biopsies compared to healthy controls. Together, these findings identify impaired oxidative and lipid metabolic programs as a key molecular feature of LGMDR1 and support the hypothesis that these metabolic defects contribute to disease pathogenesis.

**Table 2:** Mitochondrial and lipid metabolism pathways downregulated in LGMDR1 patients (identified by Metascape analysis).

Term	Description	LogP	Genes
GO:0140053	mitochondrial gene expression	−4.592	FOXO3,MRPL12,SHMT2,MTRF1,MRPL19,LRPPRC,MRPS30, MRPS27,LARS2,MRPS18B,MTERF3,TRNT1,MRPL37, MRPL48,IARS2,FASTKD5,MRPS9,MRPS5,MRPL38,MRPL34, MRPL1,MRPS34,RCC1L,MRPL45,GFM1,MALSU1,PPARGC1B, RPUSD3
GO:0046320	regulation of fatty acid oxidation	−3.572	ACADL,FABP3,PPARGC1A,MLYCD,PLIN5,CYP4A11,EPHX2, HTR2A,MVK,ABCB4,STAR,VDR,MTMR1,SOCS7,G0S2,MLXIPL, WNT4,ANGPTL8,TTC39B,DGKG,IMPA2,LCAT,DECR2,CLN8, ST3GAL5,LPIN1,NSDHL,ACSM5,PTDSS2,IDI2,SGPP2,ASAH2B, CRPPA,LHCGR,POMC,AZIN1,SELENOS
GO:0019216	regulation of lipid metabolic process	−3.287	ACADL,CYP4A11,EPHX2,FABP3,HTR2A,MVK,ABCB4,STAR,VDR,MTMR1,PPARGC1A,MLYCD,SOCS7,G0S2,MLXIPL,WNT4, ANGPTL8,TTC39B,PLIN5
GO:0046889	positive regulation of lipid biosynthetic process	−2.884	FABP3,HTR2A,STAR,PPARGC1A,MLXIPL,WNT4,PLIN5
GO:0008610	lipid biosynthetic process	−2.449	ACADL,EPHX2,CLN8,FABP3,HTR2A,LCAT,MVK,STAR,VDR,MTMR1,ST3GAL5,PPARGC1A,LPIN1,MLYCD,DECR2,NSDHL,MLXIPL,WNT4,ACSM5,PTDSS2,IDI2,SGPP2,PLIN5,ASAH2B,CRPPA
GO:0045923	positive regulation of fatty acid metabolic process	−2.240	FABP3,HTR2A,STAR,PPARGC1A,MLYCD,MLXIPL,WNT4,PLIN5
GO:0046890	regulation of lipid biosynthetic process	−2.233	ACADL,FABP3,HTR2A,MVK,STAR,VDR,PPARGC1A,MLXIPL,WNT4,PLIN5
R-HSA-1989781	PPARA activates gene expression	−3.408	ACADM,CPT2,ACSL1,HMGCS1,ME1,NRF1,RXRA,TXNRD1, NR1H2,NCOA3,PEX11A,MED27,NCOR2,MED13,GRHL1,MED8
GO:0006091	generation of precursor metabolites and energy	−3.323	ADH6,ALDH2,CALM1,CDK1,COX11,NIPSNAP2,GPD2,HK2,IDO1,INSR,MAOB,ME1,NRF1,PFKFB2,PGM1,PHKB,PIK3CA,PPP1CB,PPP1R3C,PRKAA2,PYGM,TAZ,TXNRD1,VCP,ALDH5A1,NR4A3,SLC25A12,HDAC4,PPIF,SLC25A13,ECD,NUP210,SIRT3,NNT,DMGDH,CYB5R4,SDHAF2,ACSS2,TIGAR,FASTKD3,ACSS3,IMMP2L,PNPT1,PPP1R3E,SDHAF4,AK9
R-HSA-556833	metabolism of lipids	−2.869	CISH,GPLD1,PIK3C2A,PIK3C2B,PIK3CA,TAZ,ZP3,ALDH5A1, ABHD16A,PIP5K1B,PLA2G6,PLA2G4C,SYNJ1,BPNT1,CEPT1, SMG1,RAB38,PISD,INPP5J,CHPT1,LPIN3,MPPE1,PIGZ,FAR1, SERAC1,SELENOI,TMEM150A,GPAT2,LIPH,FAM126B,SERINC2,ACADM,CPT2,DHCR7,DHCR24,ACSL1,B4GALNT1,GPD2, HMGCS1,HSD17B3,LSS,ME1,NRF1,PPP1CB,PRKAA2,RXRA, SLC22A5,ELOVL4,TXNRD1,NR1H2,NCOA3,PEX11A,MED27, NCOR2,MED13,PTGES3,NEU3,ACOT11,GRHL1,SAR1B, MTMR10,PNPLA2,ACSS3,ACSF2,SBF2,MED8,OSBPL1A, OSBPL9,TECRL,NUDT7,PNPLA7,ACOT1,SIRT1

**Table 3:** Stress-response pathways downregulated in LGMDR1 patients (identified by Metascape analysis).

Term	Description	LogP	Genes
R-HSA-2262752	Cellular responses to stress	−5.084	NUDT2,ARNT,ATM,CAMK2B,CAMK2G,CAT,CDKN1B,DYNC1H1, PHC1,ETS2,FKBP4,MTOR,H12,H2BC5,HMGA1,HSF1,HSPA9, HSP90AA1,HSP90AB1,NUP98,MAP2K7,PSMA7,PSMC2,PSMC3, PSMD7,PSMD11,RANBP2,ST13,TFDP1,HIRA,UBB,UBE2D2, CUL2,CDC16,BAG3,NUP153,DNAJB6,RAD50,TUBB4A, TXNRD2,STIP1,PSME4,TXN2,ANAPC15,TNRC6A, DYNC1LI1,CYCS,EGLN1,DNAJA4,HIF1AN,NDC1,MLST8, ANAPC1,ACD,NOX5,EHMT1,HSPA12B,ANAPC16
R-HSA-3371556	Cellular response to heat stress	−4.055	ATM,CAMK2B,CAMK2G,FKBP4,MTOR,HSF1,HSPA9,HSP90AA1,HSP90AB1,NUP98,RANBP2,ST13,BAG3,NUP153,DNAJB6,NDC1,MLST8,HSPA12B
R-HSA-3371571	HSF1-dependent transactivation	−3.624	CAMK2B,CAMK2G,FKBP4,MTOR,HSF1,HSP90AA1,HSP90AB1, MLST8

**Figure 5:**
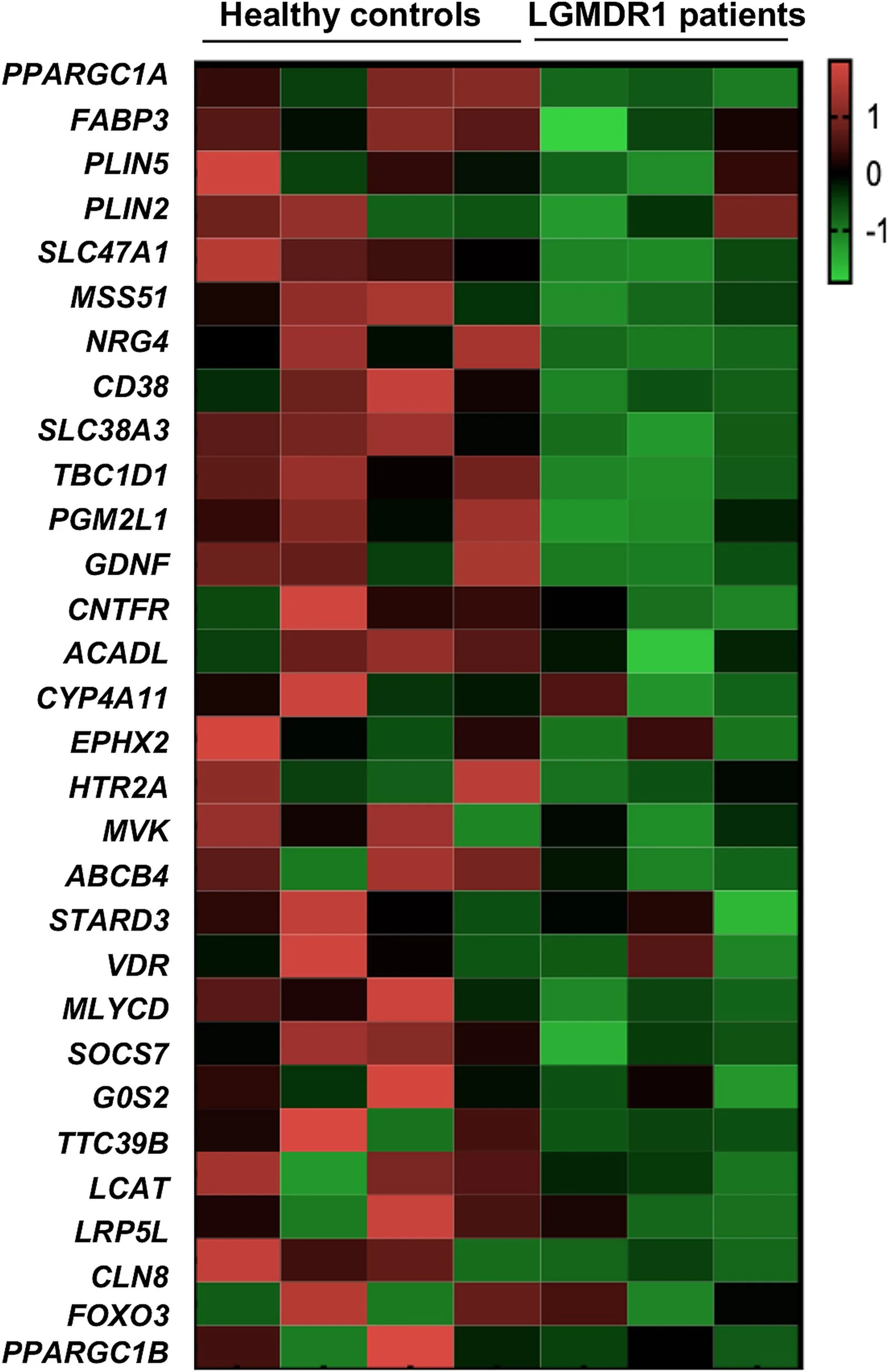
Bulk RNAseq of LGMDR1 patient muscle biopsies reveals reduced expression of metabolic genes. Heat map of bulk RNAseq data from quadriceps of 4 healthy control subjects (16–34 years old) and 3 LGMDR1 patients (17–22 years old). Color scale on the right shows the z score.

### Overexpression of constitutively active *Camk2b* increases mitochondrial respiration in C3KO muscles and improves functional performance

We next investigated whether increasing levels of active CaMKIIβ in skeletal muscle can improve the C3KO phenotype. To achieve this goal, the cDNA encoding a constitutively active mutant of *Camk2b* (CaMKII-T287D) under the control of the human skeletal actin promoter (HSA) was systemically delivered to skeletal muscle using adeno associate virus AAV serotype Rh74. This promoter is restricted to skeletal muscle and is not active in the heart [34]. To identify the dose for the study, we injected 2-month-old C3KO mice retro-orbitally with 5E13 vg/kg or 2E14 vg/kg of Rh74-CaMK2b-T287D and assessed levels of CaMKIIβ in various skeletal muscles 6 weeks post injection. As shown in Supplemental Fig. 5, a substantial increase in CaMKIIβ levels was observed in C3KO skeletal muscles injected with 2E14 vg/kg Rh74- CaMK2b-T287D compared to C3KO mice injected with PBS or 5E13vg/kg. This treatment was well tolerated by the mice; no lethality or serious health problems were observed and suggested a dose of 2E14 vg/kg for the subsequent study.

For the long-term studies, 10-week-old C3KO mice (n = 8 males per group) were injected with either 2E14 vg/kg of Rh74-*CaMK2b*-T287D or the same dose of empty Rh74 as a control. Mice were tested every 5 weeks after the injection to assess functional performance using the wire mesh test. As shown in Fig. 6, C3KO injected with Rh74-*Camk2b*-T287D showed a trend towards improved functional performance starting 5 weeks post dosing compared to C3KO mice injected with empty Rh74. Significant strength increases were observed in treated mice from 20 to 35 weeks post dosing. Histological examination of skeletal muscles and hearts from Rh74-*Camk2b-*T287D—injected mice did not reveal any pathological changes (Supplemental Fig. 6).

**Figure 6:**
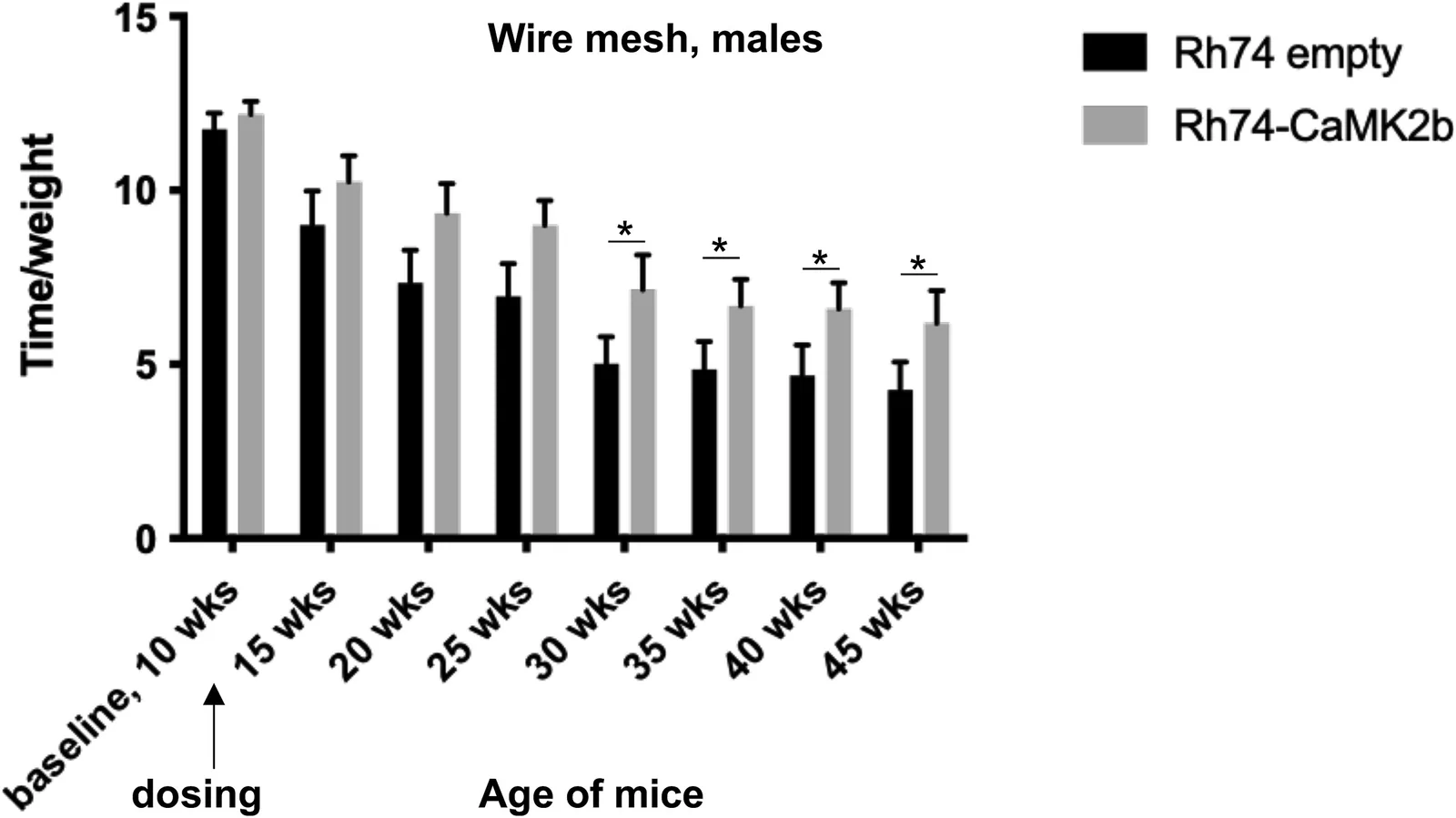
Expression of constitutively active *Camk2b* in C3KO skeletal muscles after systemic delivery by AAV-Rh74 improves functional performance. C3KO mice were functionally tested by wire mesh at baseline (at 10 wks of age); then dosed the next day with Rh74-CaMK2b-T287D (2E14 vg/kg) or Rh74 empty (2E14 vg/kg) and tested every 5 weeks after dosing until they reached 45 weeks of age. Error bars represent standard deviation; paired *t-*test was used for statistical analysis (n = 8 for each group), ^*^  *P* < 0.05 was considered statistically significant.

C3KO mice have decreased muscle weight (muscle atrophy) and reduced slow myofiber phenotype and both features likely contribute to the impaired muscle performance observed in this model of LGMDR1 [18]. As shown in Supplemental Fig. 7, overexpression of constitutively active CaMK2β had no effect on muscle weight or fiber cross-sectional area despite the improved muscle performance.

Since CaMKII signaling promotes the oxidative phenotype in skeletal muscle, we assessed the oxidative capacity of soleus and plantaris muscles from dosed C3KO mice by Seahorse analysis or NADH staining. These data revealed that the maximal oxidative rate was significantly higher in the Rh74-*CaMK2b*-T287D injected group compared to the control group for mitochondrial complex II and fatty acid oxidation (Fig. 7A).

**Figure 7:**
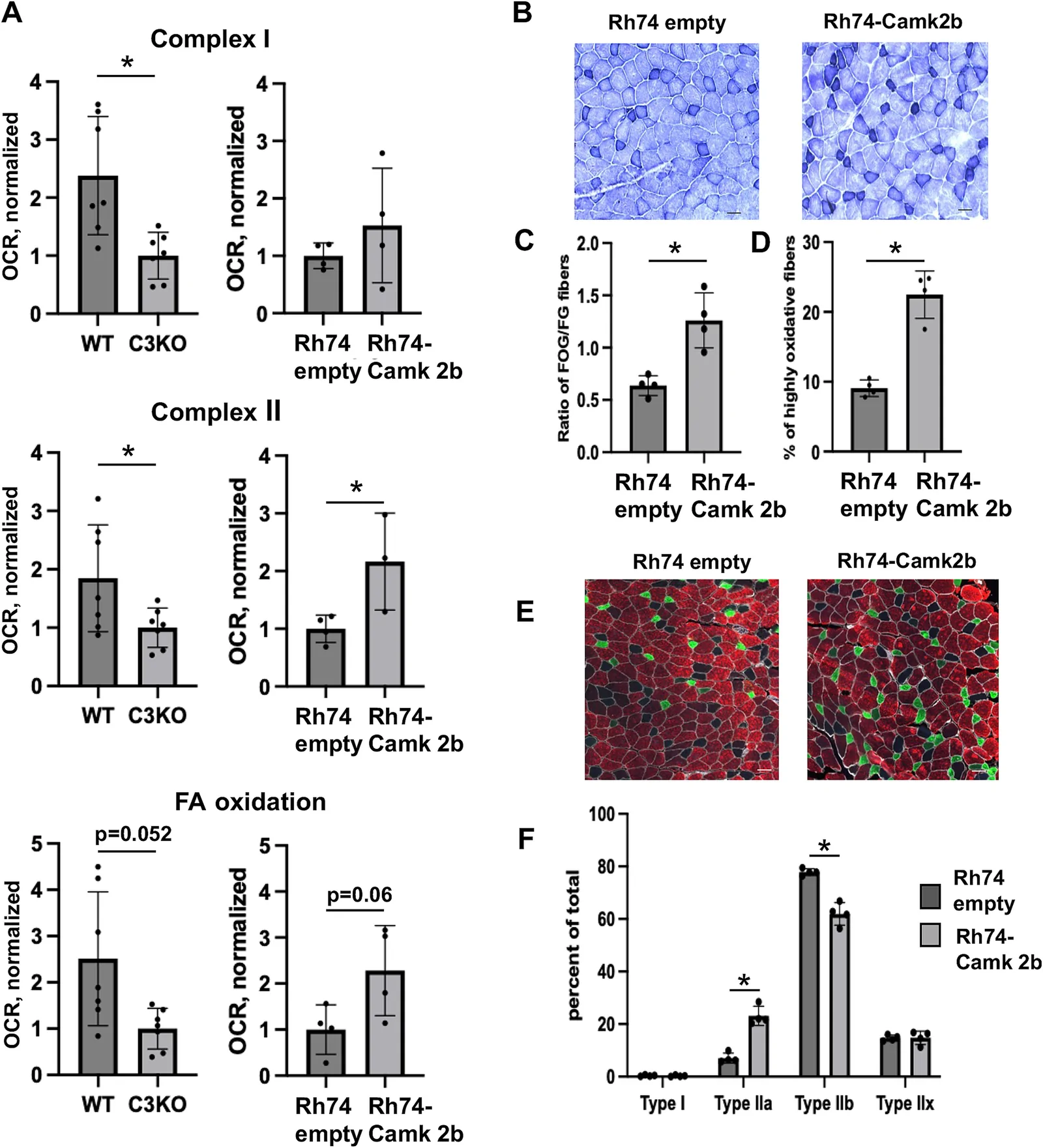
Overexpression of constitutively active *Camk2b* improves oxidative capacity of C3KO skeletal muscles. (A) Mitochondrial respiration assayed by Seahorse in mitochondrial fractions from freshly dissected soleus muscles from 8-month-old C3KO or WT mice (left panels), and Rh74-*CaMK2b*- or Rh74 empty-injected mice (right panels). Maximal oxidative rate was determined using three different substrates to assess complex I, complex II, and fatty acid oxidation. (B) Histological images of plantaris muscles of Rh74-CaMK2b- or Rh74 empty-injected mice stained for NADH activity. Three categories of stained fibers were identified based on their oxidative capacity; dark and intermediate blue fibers represent slow and fast oxidative fibers (FOG) while light blue fibers represent low oxidative mostly glycolytic fibers (FG). (C and D) Quantitative analysis of NADH stained plantaris muscle sections showing the ratio of FOG/FG fibers (C) and the percentage of highly oxidative (dark blue) fibers (D) in Rh74-CaMK2b- or Rh74 empty-injected mice. (E) Representative images of plantaris muscles from Rh74-*CaMK2b*- or Rh74 empty-injected mice used for fiber type analysis (type I fibers are blue, type. IIA fibers are green, type IIB fibers are red and type IIX fibers are black). (F) Quantitative analysis of the fiber type composition in plantaris muscles of Rh74-CaMK2b- or Rh74 empty-injected mice. Error bars represent standard deviation; ^*^P < 0.05 was considered statistically significant by two tailed *t-*test. Scale bar in B and E, 50 mμ.

The muscle oxidative capacity in Rh74-*Camk2b*-T287D injected C3KO mice was also evaluated by NADH staining of the plantaris muscles (Fig. 7B and C) showing that the percent of highly oxidative slow muscle fibers (stained dark blue) and the ratio of fast oxidative/glycolytic (FOG) to fast glycolytic fiber (FG) fibers were significantly higher in mice injected with Rh74-*Camkb*-T287D compared to controls. We performed fiber analysis in plantaris muscles of Rh74-*Camk2b*-T287D injected and control mice (Fig. 7E and F). This assessment revealed an increase in the percent of the type IIA (FOG) fibers with a concomitant reduction in the percent of type IIB (FG) fibers after dosing C3KO mice with Rh74-*Camk2b*-T287D. This effect was somewhat unexpected, as we did not observe changes in MyHC isoform expression in CaMK2b cKO mice (Fig. 2B). It is possible that constitutively active CaMKIIβ can activate other forms of CaMKII due to ability of CaMKII to form hetero-oligomeric complexes [35] which may have enhanced the effect of CaMKIIβ overexpression. These results strongly suggest that restoring CaMKIIβ signaling can increases oxidative capacity and improve muscle function in the mouse model of LGMDR1.

Taken together, these data show that CaMKIIβ-mediating signaling promotes oxidative metabolism in skeletal muscle and is important for endurance exercise adaptation. Downregulation of this pathway in C3KO mice significantly contributes to compromised slow oxidative gene expression and metabolism. RNA-seq data also revealed a deficiency in metabolic gene expression in LGMDR1 patients compared to age-matched healthy control subjects, providing support for the hypothesis that metabolic insufficiency is an underlying feature of LGMDR1. Finally, the data suggest that increasing CaMKIIβ signaling in C3KO mice can reverse several aspects of the calpainopathy phenotype making it a possible target for therapeutic intervention to treat LGMDR1.

## Discussion

The goal of these studies was to define the contribution of blunted CaMKIIβ signaling to the calpainopathy phenotype described in previous publications [6, 8]. CaMKIIβ-mediated signaling has been shown to regulate several processes that control skeletal muscle gene expression. Early studies showed that CaMKII signaling promotes expression of genes encoding slow isoforms by increasing activation of the transcription factor MEF2 [1, 36, 37] and that CaMKII activation is required for efficient muscle regeneration following injury [38]. All four CaMKII isoforms are expressed in skeletal muscles; however, the specific function of each individual isoform and the impact on gene expression in response to loading have not been investigated.

Our RNA-seq analysis identified two CaMKII isoforms, encoded by *Cam2a* and *Camk2b genes*, as being expressed at relatively high levels in murine skeletal muscle, with *Camk2b* emerging as the dominant isoform in soleus (unpublished data). This is noteworthy because the soleus is one of the few mouse muscles with a substantial proportion of slow-twitch fibers, in contrast to human skeletal muscle, which generally exhibits a more balanced distribution of fast and slow fibers. The prominence of *Camk2b* in soleus, coupled with the fact that soleus is among the most severely affected muscles in C3KO mice [18], highlights the possibility that CaMKIIβ activity may be especially important for maintaining the integrity and metabolic function of slow fibers.

Prior studies have suggested that slow fibers are disproportionately affected in LGMDR1, although direct correlations between fiber type and disease indices remain limited in human biopsy datasets. Nonetheless, a consistent feature across multiple reports is the enrichment of lobulated fibers in LGMDR1 biopsies and mitochondrial dysfunction [6, 39–41]. The observation that lobulated fibers appear to be predominantly in slow muscles lends support to the notion that slow fibers are more affected than fast in LGMDR1 [6, 39–41]. The predominance of lobulated fibers in slow muscles has two important implications. First, it suggests that the pathological process in LGMDR1 is not uniformly distributed across fiber types but instead reflects a selective vulnerability of slow fibers. Second, it underscores the likelihood that metabolic defects, rather than gross structural abnormalities alone, contribute significantly to disease progression. Taken together with our findings in C3KO mice, these observations strengthen the argument that impaired CaMKIIβ signaling and downstream metabolic regulation disproportionately impact slow oxidative fibers, thereby shaping the muscle-specific phenotype of LGMDR1.

Our previous studies demonstrated that blunted CaMKII-mediated signaling in C3KO muscles coincided with impaired muscle adaptation [6, 8, 28]. In those studies, we utilized two experimental models of muscle adaptation: endurance running exercise and muscle regrowth following a period of disuse [8, 28]. In wild-type muscles, CaMKII activation was strongly correlated with muscle loading in both experimental conditions. However, in C3KO muscles, CaMKII activation was severely blunted, which was associated with defective gene expression and impaired muscle adaptation [6, 8, 28]. During endurance running, three major gene groups failed to be induced in C3KO mice: 1) metabolic genes involved in oxidative metabolism, particularly those regulating β-oxidation of fatty acids; 2) genes encoding slow isoforms of myofibrillar proteins and 3) stress response genes. Although these deficits were strongly associated with blunted CaMKII signaling, it remained unclear whether all the observed impairments were directly caused by CaMKII dysfunction or if additional factors were involved. While *Camk2b* knockout mice were previously generated, phenotypic studies were mostly limited to studies of brain function [29]. Interestingly, it was shown that besides a neuronal defect, *Camk2b* knockout mice also demonstrated decreased forelimb grip strength [29]. These data prompted us to generate a muscle-specific conditional knockout of *Camk2b* to investigate the unique role of this isoform in skeletal muscles.

Data presented here show that CaMKIIβ is indispensable for normal muscle function and its absence can’t be fully compensated by other CaMKII isoforms. *Camk2b* cKO mice performed significantly worse than their control littermates in all three functional tests that we used. However, histological evaluation revealed relatively mild morphological changes with a variable, but higher than normal presence of centrally nucleated fibers while no significant degeneration and regeneration was observed in muscles of young (1–2 months of age) or old (6–7 months of age) mice. The same phenotypic features have been reported in C3KO mice and have been observed to differ from skeletal muscle phenotypes for other forms of muscular dystrophy such as the mdx model of Duchenne muscular dystrophy, which shows extensive degeneration and regeneration [18].

Even though CaMKII signaling has been shown to promote the slow phenotype, we did not observe statistically significant changes in the percent of slow or FOG fibers in *Camk2b* cKO muscles compared to control mice (Fig. 2). Furthermore, we did not observe abnormalities in transcription of sarcomeric genes induced by running exercise in the *CamkIIb* cKO, suggesting that other isoforms of CaMKII may have compensated for the lack of CaMKIIβ to induce slow isoforms of myofibrillar genes. This hypothesis is indirectly supported by the fact that overexpression of constitutively active CaMKIIβ that can activate other CaMKII isoforms, promotes a shift from glycolytic type IIB fibers toward more oxidative type IIA in C3KO mice (Fig. 7F) Other Ca^2+^-mediated signaling pathways may also contribute to regulation of sarcomeric genes. Identifying these pathways, including their downstream transcription factors, as well as further characterizing the functional consequences of CaMKIIβ overexpression (such as by assessing Ca^2+^ handling and directly measuring muscle force and fatigability) will be important goals for future studies.

Our data showed that CaMKIIβ-mediated signaling promotes expression of genes involved in lipid metabolism and fatty acid oxidation, suggesting that the blunted CaMKIIβ signaling likely underlies the lipid metabolism defect previously observed in C3KO. In skeletal muscle, fatty acid oxidation plays an important role as a source of energy especially during prolonged muscle activity [42]. Even though gene signatures of *Camk2b* cKO and C3KO shared many similarities, the deviations from the controls were stronger in C3KO mice compared to *Camk2b* cKO mice. These data suggest that CAPN3 acts upstream of CaMKIIβ and affects other pathways. Data from our lab and from others indicate that CAPN3 is required for normal Ca^2+^ trafficking and homeostasis [7, 9, 43]. We found that CAPN3 is enriched in the triad fraction and that the lack of CAPN3 is associated with destabilization of the triad protein complex, reduced levels of triad-associated CaMKII and decreased Ca^2+^ release upon excitation in young (2 month) presymptomatic and old (1 year) C3KO mice [7, 9]. Given the multiple Ca^2+^-dependent pathways that operate in muscle, all of which could potentially be affected by the observed Ca^2+^ abnormalities, we cannot rule out involvement of these pathways in LGMDR1 pathogenesis. In our previous studies we showed that calcineurin levels, AKT signaling and AMPK activation were not changed in C3KO muscles [8, 24, 27]; however, more in depth studies are required to elucidate how the absence of CAPN3 impacts other Ca^2+^ signaling pathways, gene expression and muscle adaptation.

Our previous studies in C2C12 myoblast cell cultures and in C3KO mice suggested that CaMKII signaling controls expression of stress response genes, particularly *Hspa1a* and *Hspa1b* [28]. Both genes encode an inducible HSP70 that acts not only intracellularly as a molecular chaperone but can also be secreted as a Damage Associated Molecular Pattern (DAMP) molecule [33]. DAMPs play an important protective role in cells by inducing an appropriate systemic response to stressful conditions such as exercise or muscle reloading after a period of disuse. Previously, we found that induction of *Hsp1a* and *Hsp1*b as well as other stress response genes was significantly reduced in C3KO muscles compared to WT muscles in both experimental settings [28]. We also showed that the systemic inflammatory response that normally accompanies muscle recovery after a period of disuse, was compromised in C3KO mice affecting recruitment of inflammatory cells needed for muscle regeneration and ultimately affecting muscle growth in these mice. Here we found that similar to C3KO muscle, stress response genes were not upregulated in *Camk2b* cKO to the same extent as controls. These data corroborate our previous observations and demonstrate that CaMKIIβ-mediated signaling activates inducible HSP70 genes in the setting of muscle remodeling.

The RNA sequencing data from human biopsies presented here provide the first evidence that genes involved in oxidative metabolism are downregulated in young LGMDR1 patients. While the metabolic aspects of LGMDR1 have not been systematically studied, previous reports have documented mitochondrial abnormalities and reduced activity of mitochondrial respiratory chain complexes in LGMDR1 muscle biopsies [14, 44, 45]. Additionally, we and others have demonstrated mitochondrial abnormalities and dysfunction in C3KO cells and mice [19, 46]. These data suggest that metabolic insufficiency could be a primary pathological feature underlying calpainopathy, leading to inadequate energy production and the inability of muscle to meet functional requirements. Further studies are necessary to corroborate this hypothesis and to identify molecular pathways involved in this process.

## Materials and methods

### Animals

All experimental protocols and the use of animals were in accordance with the National Institutes of Health Guide for Care and Use of Laboratory Animals and approved by the UCLA Institutional Animal Care and Use Committee. *Camk2b*^fl/fl^ mouse line was generated at the Institut Clinique de la Souris (France) and was described previously [29]. Frozen embryos for this line were obtained from the Centre European de Recherche en Biologie et Medecine (France). HSA-cre transgenic mice were obtained from the Jackson Laboratories. C3KO mice were previously described [18]. Both *Camk2b* cKO and C3KO mice are congenic to C57/BL6. AAV Rh74-HSA-CaMK2b-T287D and empty control AAV Rh74 (5E13 vg/kg or 2E14 vg/kg for the sort-term studies and 2E14 vg/kg for the long-term experiment) were delivered to C3KO mice by retro-orbital vein injection (n = 8 for each group). AAV was packaged, purified and tittered by qPCR by Virovek.

### Treadmill exercise and other functional tests

#### Treadmill running for gene expression studies

Male mice (*n* = 6 for each genotype, 6-month old) were accustomed to the treadmill (Columbus Instruments) with a 15 min run once per day at 10 m/min for 3 days. For the next 5 days, mice were subjected to forced running exercise performed on a 10% positive incline at 10 m/min for the first 20 min followed by 40 min at 15 m/min. Mice were sacrificed two hours after the last run. Age-matched males (*n* = 6 for each genotype) that were not subjected to exercise were used as sedentary controls.

#### Run to exhaustion

Mice were run on a treadmill on a 15% positive incline at a starting speed of 1 m/min. Speed was increased by 1 m/min every minute until the mice were unable to perform. All mice used for these experiments were males (*n* = 11–12 for each genotype), aged 6–7 months.

#### Wire mesh

Male mice were placed on the wire mesh pulled over a wooden frame on a swivel (custom built). Once a mouse was in position, the wire mesh was rotated 180°, resulting in the mouse hanging upside down from the mesh, using all four limbs. Five trials were conducted with 1 min rest between the trials. The average of all 5 attempted was used for statistical analysis. All mice used for these experiments were males (*n* = 12–13 for each genotype), aged 6–7 months.

#### Forelimb grip strength

Grip strength was measured using a digital force gauge (DFIS2, Chatillon CE). Five trials were performed with 30 sec of rest between the trials. Data were presented as an average of 5 trials. All mice used for these experiments were males (*n* = 12–13 for each genotype), aged 6–7 months.

### Human studies

Human quadriceps muscle biopsies were provided by the Iowa Wellstone MDCRC Muscle Biopsy Repository. The following samples were used for RNA seq analysis.

**Table:** 

	Diagnosis	Sex	Age at the time of biopsy, years	Pathogenic variants (transcript NM_000070.2)
1	Healthy control subject	female	17	n/a
2	Healthy control subject	male	34	n/a
3	Healthy control subject	male	16	n/a
4	Healthy control subject	female	29	n/a
5	LGMDR1 patient	male	22	c.1355-1G>C/c.759_761del
6	LGMDR1 patient	male	17	c.883delinsCTT/c.1371C>A
7	LGMDR1 patient	female	19	c.264T>G/c.2362_2363delinsTCATCT

### Histological analysis

All histological studies were performed on 10 μm frozen muscle sections. Muscle sections from 3–6 mice per group were used for histological studies. Hematoxylin and eosin staining was used to evaluate overall pathological changes including centrally nucleated fibers. For immunohistochemistry, muscle sections were thawed at room temperature and fixed with cold acetone for 10 min. Sections were blocked first with a non-specific blocking buffer (0.2% gelatin, 0.5% Tween-20 and 3% bovine serum albumin in PBS) followed by M.O.M. blocking solution (Vector Laboratories). Rat anti-mouse CD68 antibody (BD Biosciences) was used to stain inflammatory cells, mouse anti-developmental myosin heavy chain (devMyHC) (Leica Biosystems) was used to stain newly formed myofibers. Images were acquired, processed and analyzed using Axio Imager M1 microscope and Axiovision software (Zeiss).

For NADH staining, frozen sections of plantaris were air-dried at room temperature and then incubated with the staining solution (1 mg/ml Nitro Blue Tatrazolium and 1 mg/ml reduced NADH in 50 mM Tris–HCL, pH 7.4). All reagents were obtained from Sigma. After incubation, slides were washed quickly in sequential acetone solutions of 30%, 60%, 90%, 60%, 30% and in water. NADH histochemistry leads to three distinguishable levels of staining that we identified as low, intermediate and high. The three levels of staining were analyzed by a blinded evaluator to ensure objectivity. We refer to the low stained fibers as fast glycolytic (FG), and the intermediate plus high together as fast glycolytic/oxidative (FOG).

For fiber type analysis, frozen sections were fixed for 5 min in ice-cold acetone and blocked for 1 h in 1% BSA prior to overnight incubation at 4°C in-anti lama2 (rat IgG1, 1:100, Sigma L0663), myosin heavy chain Type 1 (mouse IgG2b, 1:10; BA-F8, DSHB), myosin heavy chain Type 2a (mouse IgG1, 1:30; SC.71, DSHB), and myosin heavy chain Type 2b (mouse IgM, 1:30; 6-H1, DHSB) in blocking reagent. The following day, slides were incubated for 1 h with goat anti-mouse IgG2b AF350 (A21140, Thermo Fisher Scientific), goat anti-mouse IgG1 AF488 (A21121, Thermo Fisher Scientific), goat anti-mouse IgM AF594 (A21044, Thermo Fisher Scientific), and donkey anti-rat AF647 (A78947, Thermo Fisher Scientific) before mounting with ProLong Gold Antifade mountant (P36930, Thermo Fisher Scientific). and All sections were imaged on a Keyence microscope at 20× magnification. Type 2B, type 2A, and type 1 fibers were manually quantified and expressed as a percentage of total myofibers per muscle section.

### Measurement of oxygen consumption rates(OCR) in mitochondrial fractions

For OCR analysis, freshly dissected soleus muscles were homogenized on ice in a Dounce homogenizer in Chappell-Perry buffer (100 mM KCl, 50 mM Tri-HCl, 5 mM MgCl_2_, 12 mM EGTA, adjusted to pH 7.4 with KOH) supplemented with 1 mM ATP and 0.5% fatty acid-free (Fraction V) BSA immediately before use. Muscle homogenates were centrifuged at 600 x g for 10 min at 4°C. Supernatants were transferred to new tubes and then centrifuged at 10 000 g for 10 min at 4°C to pellet mitochondria. Mitochondria were resuspended in SHE buffer (250 mM sucrose, 5 mM HEPES, 1 mM EGTA, adjusted to pH 7.2 with KOH). Protein concentrations were measured using BCA protein Assay Kit (Thermo Fisher Scientific). The samples (4 μg/well) were analyzed in the UCLA Mitochondrial and Metabolism Core using a Seahorse XF96 Analyzer (Agilent). Pyruvate plus malate were used to assess Complex I-dependent respiration, succinate plus rotenone were used to assess Complex II-dependent respiration, and palmitoyl-carnitine was used to assess fatty acid oxidation*.* Data were normalized to total protein. Seahorse analysis was carried out according to Acin-Perez *et al*. [47].

### Western blot analysis

Muscles were homogenized by hand in a Dounce homogenizer in reducing sample buffer (80 mM Tris, pH 6.8, 10% β-mercaptoethanol, 2% SDS and 10% glycerol) with Halt phosphatase and protease inhibitor cocktail (Thermo Fisher Scientific). An equal amount of total protein was loaded on SDS-PAGE gels followed by transfer to nitrocellulose membrane. Ponceau red S was used to verify the transfer onto the nitrocellulose. The following primary antibodies were used for western blotting: anti-CaMKII (1:500, Cell Signaling, D11A10), anti-phospho-CaMKII alpha/beta (Thr286) (1:1000, ThermoFisher PA1-4614), anti-CaMKII beta (1:500, ThermoFisher 13-9800) anti-hsp70 (StressMarq), anti-hsp90 (BD Transduction Laboratories). Secondary antibodies conjugated with HRP were from Sigma-Aldrich (used 1:10000). Specific signals were developed using ChemiGlow chemiluminescent substrate for HRP (Protein Simple). Images of the blots were acquired using FluorChem FC2 Imager (Alpha Innotech). Specific signals were developed using ChemiGlow chemiluminescent substrate for HRP (Protein Simple). Images of the blots were acquired using FluorChem FC2 Imager (Alpha Innotech). Quantitative analysis was performed using ImageJ software.

### RNA-Seq

RNA-seq was performed at the UCLA Technology Center for Genomics & Bioinformatics.

Libraries for RNA-Seq were prepared with KAPA mRNA-Seq Hyper Prep Kit. The workflow consists of mRNA enrichment and fragmentation, first strand cDNA synthesis using random priming followed by second strand synthesis converting cDNA:RNA hybrid to double-stranded cDNA (dscDNA) to incorporate dUTP into the second cDNA strand. cDNA generation is followed by end repair to generate blunt ends, A-tailing, adaptor ligation and PCR amplification. Different adaptors were used for multiplexing samples in one lane. Sequencing was performed on Illumina NovaSeq6000 for a PE 2 x 50 run. Data quality check was done on Illumina SAV. Demultiplexing was performed with Illumina Bcl2fastq v2.19.1.403 software. The reads were mapped by STAR 2.7.9a [48] and read counts per gene were quantified using the mouse genome mm10.102 or human genome GRCh38. In Partek Flow (Partek® Flow® software, v7.0 Copyright ©. 2019 Partek Inc., St. Louis, MO, USA), read counts were normalized by median ratio for use in DESeq2 [49]. All results of differential gene expression analysis utilized the DESeq2 statistical analysis tool [49]. *P*-value (< 0.05) and fold change (> 2×) filters were applied for differentially expressed gene lists prior to downstream analysis.

### Statistical analysis

For all studies, results from quantitative experiments were expressed as means ± standard deviation. Statistical significance was calculated by Student’s t test (for two group comparisons), paired t test (for repeat measures in two groups), one-way ANOVA (for more than two groups comparison); p value less than 0.05 was considered statistically significant.

## Supplementary Material

Supplementary_material_ddag090
